# Comprehensive behavioral phenotyping of male *Septin 3*-deficient mice reveals task-specific abnormalities

**DOI:** 10.1186/s13041-025-01243-5

**Published:** 2025-08-22

**Authors:** Natsumi Ageta-Ishihara, Keizo Takao, Tsuyoshi Miyakawa, Makoto Kinoshita

**Affiliations:** 1 Department of Biomolecular Science, Faculty of Science, Toho University, 2-2-1 Miyama, Funabashi, 274-8510 Chiba Japan; 2 Department of Molecular Biology, Division of Biological Sciences, Nagoya University Graduate School of Science, Chikusa-ku, Nagoya, 464-8602 Japan; 3 Department of Behavioral Physiology, Faculty of Medicine, University of Toyama, Toyama, 930-0194 Japan; 4 Center for Genetic Analysis of Behavior, National Institute for Physiological Sciences, Okazaki, Aichi, 444-8585 Japan; 5 Division of Systems Medical Science, Center for Medical Science, Fujita Health University, Toyoake, Aichi, 470-1192 Japan

**Keywords:** Septin, Knockout mice, Behavioral phenotyping, Social interaction, Contextual fear conditioning, Spatial working memory

## Abstract

The septin cytoskeleton is recognized as the fourth component of the cytoskeleton. Septin 3 (SEPT3)/G-septin is a neuron-selective subunit of the septin family and is widely expressed in mature neurons. We previously demonstrated that SEPT3 regulates long-term potentiation (L-LTP)-dependent extension of smooth endoplasmic reticulum (sER) into dendritic spines of granule cells in the hippocampal dentate gyrus (DG), and that *Sept3* knockout (*Sept3*^−/−^) mice exhibited impairments in DG-dependent spatial long-term memory. However, the broader behavioral consequences of SEPT3 deficiency remain largely unexplored. To address this, we conducted comprehensive behavioral phenotyping of male *Sept3*^−/−^ mice using a standardized test battery. In the social interaction test in a novel environment, *Sept3*^−/−^ mice showed increased contact frequency and interaction time. In contrast, performance in the three-chamber social interaction test was comparable to wild-type mice, indicating context-dependent deficits. In contextual fear conditioning, *Sept3*^−/−^ mice displayed reduced freezing 24 h after training, but not 35 days later. In the T-maze forced alternation task, deficits were observed in choice accuracy and latency. These results demonstrate that *Sept3*^−/−^ mice exhibit selective behavioral impairments depending on task demands and environmental context. Our findings provide the first behavioral characterization of *Sept3*^−/−^ mice and offer new insights into the functional role of SEPT3, laying a foundation for future investigation into its molecular and circuit-level mechanisms.

**Supplementary Information:**

The online version contains supplementary material available at 10.1186/s13041-025-01243-5.

## Main text

The septin cytoskeleton comprises guanosine triphosphate (GTP)-binding proteins that assemble into hetero-oligomeric complexes, recognized as the fourth cytoskeletal component, following actin filaments, microtubules, and intermediate filaments [1, 2]. Among the thirteen known mammalian septin genes (SEPT1 to SEPT12 and SEPT14), SEPT3/G-septin is a brain-specific member of the septin family with prominent expression in postmitotic neurons [3]. SEPT3 is expressed in pyramidal neurons in the hippocampal cornu ammonis 1 (CA1)–CA3 subfields, granule cells of the hippocampal DG, the cerebral cortex, and the cerebellum [3–5]. In addition, SEPT3 has been implicated in neuronal autophagy [6] and has also been associated with Alzheimer’s disease [7, 8]. Previous studies using *Sept3*^−/−^ mice have shown that overall neuronal architecture remains grossly intact, with normal morphology observed in cultured hippocampal pyramidal neurons and in brain regions such as the CA1 area, cortex, and cerebellum [5, 9]. Electrophysiological analyses have likewise demonstrated unaltered basal synaptic transmission at CA3–CA1 synapses [9].

Recently, we have shown that SEPT3 promotes L-LTP-dependent extension of smooth endoplasmic reticulum into dendritic spines of DG granule cells, enhancing Ca^2+^ signaling and synaptic efficacy. Moreover, it contributes to DG-dependent spatial long-term memory, while short-term memory remains unaffected [4, 10]. Despite its broad expression in mature neurons throughout the brain, the contribution of SEPT3 to behavior across a broader range of functional domains has not been systematically examined. In this study, we performed comprehensive behavioral phenotyping of *Sept3*^−/−^ mice.

Experimental animals were obtained by intercrossing *Sept3*^+/−^ heterozygotes [4], yielding male *Sept3*^−/−^ mice and wild-type (*Sept3*^+/+^) mice. All animals were housed under a 12-h light/dark cycle, with ad libitum access to food and water. Behavioral tests were conducted according to our previously described standardized protocols [11–13]. Statistical analyses were performed using Prism (GraphPad Software) with two-tailed unpaired *t* test or two-way repeated measures ANOVA.

General health, neuromuscular function, motor abilities, and sensory function were first assessed to exclude potential confounding factors in behavioral analysis. *Sept3*^−/−^ mice showed no significant differences from *Sept3*^+/+^ mice in body weight, rectal temperature, wire hang latency, grip strength, rotarod performance, or latency to respond in the hot plate test (Fig. S1–S3). These results indicate that basic physiological condition, motor abilities, and pain sensitivity were preserved in *Sept3*^−/−^ mice.

**Fig. 1:**
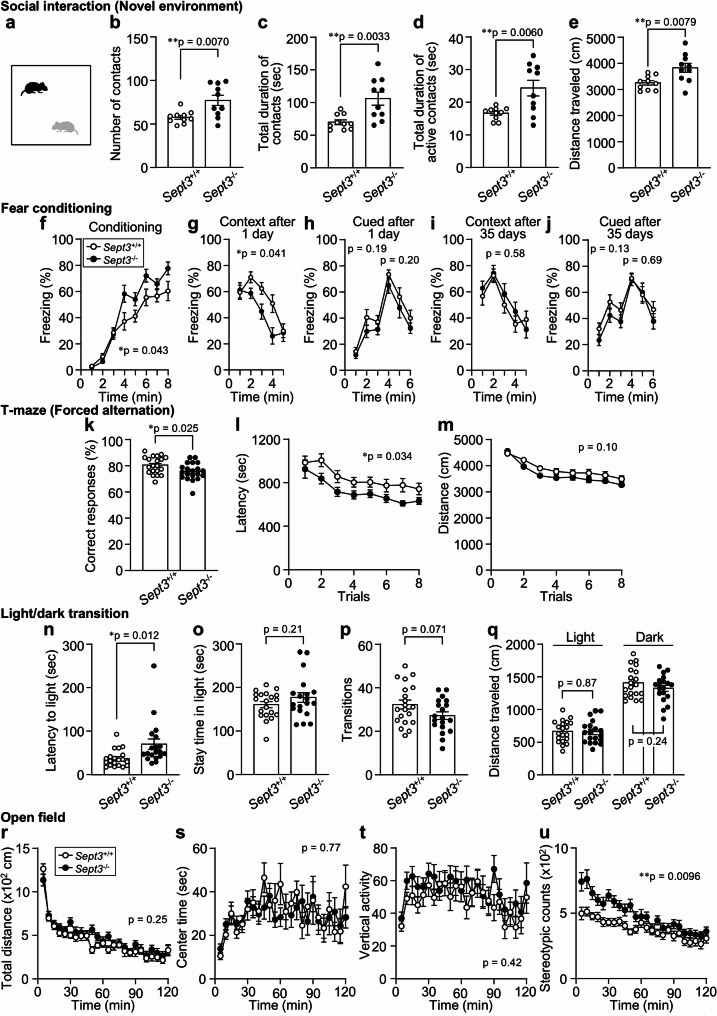
Behavioral alterations in *Sept3*^−/−^ mice across selected social and cognitive tasks **a–e**, Social interaction test in a novel environment (single-chamber). **a**, Schematic of single‑chamber social approach test in a novel environment. **b**, Number of contacts. **c**, Total duration of contacts. **d**, Total duration of active contacts. **e**, Distance traveled. *n* = 10 (*Sept3*^+/+^) and *n* = 10 (*Sept3*^−/−^) 12–14-week-old male mice pairs; two-tailed unpaired *t* test. **f–j**, Contextual and cued fear conditioning test. **f**, Freezing during acquisition of the association between a foot shock and a preceding auditory cue (tone) in context A [F_1,37_ = 4.39, *p* = 0.043, genotype × time interaction, F_7,259_ = 3.20, *p* = 0.0029]. **g**, **i**, Freezing in context A without the cue, tested 1 (**g**) or 35 (**i**) days after conditioning [1 day, F_1,37_ = 4.49, *p* = 0.041, genotype × time interaction, F_4,148_ = 2.76, *p* = 0.03, 35 days, F_1,37_ = 0.32, *p* = 0.58, genotype × time interaction, F_4,148_ = 1.01, *p* = 0.41]. **h**, **j**, Freezing in response to the tone in context B, tested 1 (**h**) or 35 (**j**) days after conditioning [1 day 1–3 min, F_1,37_ = 1.76, *p* = 0.19, genotype × time interaction, F_2,74_ = 0.68, *p* = 0.51, 1 day 4–6 min, F_1,37_ = 1.69, *p* = 0.20, genotype × time interaction, F_2,74_ = 0.0049, *p* = 1.00, 35 days 1–3 min, F_1,37_ = 2.41, *p* = 0.13, genotype × time interaction, F_2,74_ = 0.034, *p* = 0.97, 35 days 4–6 min, F_1,37_ = 0.17, *p* = 0.69, genotype × time interaction, F_2,74_ = 1.01, *p* = 0.37]. *n* = 20 (*Sept3*^+/+^) and *n* = 19 (*Sept3*^−/−^) 56–58-week-old (**f**–**h**) or 62–64-week-old (**i**, **j**) male mice; two-way repeated measures ANOVA. **k–m**, T-maze forced alternation test. **k**, Correct responses; two-tailed unpaired *t* test. **l**, Total duration of the trial [F_1,38_ = 4.82, *p* = 0.034, genotype × time interaction, F_7,266_ = 0.58, *p* = 0.77]; two-way repeated measures ANOVA. **m**, Total distance traveled during the trial [F_1,38_ = 2.78, *p* = 0.10, genotype × time interaction, F_7,266_ = 1.43, *p* = 0.19]; two-way repeated measures ANOVA. *n* = 20 (*Sept3*^+/+^) and *n* = 20 (*Sept3*^−/−^) 28–30-week-old male mice. **n–q**, Light/dark transition test. **n**, Latency until the first entry into the light chamber. **o**, Time spent in the light chamber. **p**, Number of transitions across the light/dark border. **q**, Distance traveled in the light and dark chambers. *n* = 20 (*Sept3*^+/+^) and *n* = 19 (*Sept3*^−/−^) 11–13-week-old male mice; two-tailed unpaired *t* test. **r–u**, Open field test **r**, Total distance [F_1,38_ = 1.35, *p* = 0.25, genotype × time interaction, F_23,874_ = 1.56, *p* = 0.045]. **s**, Center time [F_1,38_ = 0.090, *p* = 0.77, genotype × time interaction, F_23,874_ = 1.03, *p* = 0.43]. **t**, Vertical activity [F_1,38_ = 0.67, *p* = 0.42, genotype × time interaction, F_28,874_ = 0.86, *p* = 0.65]. **u**, Stereotypic counts [F_1,38_ = 7.43, *p* = 0.0096, genotype × time interaction, F_23,874_ = 3.11, *p* = 0.0000015]. *n* = 20 (*Sept3*^+/+^) and *n* = 20 (*Sept3*^−/−^) 11–13-week-old male mice; two-way repeated measures ANOVA Data are mean ± SEM. *p < 0.05, **p < 0.01

We next examined social behavior using two complementary tests. In the social interaction test conducted in a novel environment (Fig. 1a), *Sept3*^−/−^ mice showed a greater number of contacts with the stimulus mouse compared with *Sept3*^+/+^ mice (Fig. 1b). Total and active contact durations were also increased in *Sept3*^−/−^ mice (Fig. 1c, d). In addition, distance traveled during the session was higher in *Sept3*^−/−^ mice (Fig. 1e). These findings indicate that *Sept3*^−/−^ mice engage in more frequent and prolonged social interaction under novel environmental conditions. In the three-chamber social interaction test (Fig. S4), there were no significant differences in sociability (interaction with a novel male mouse vs. an empty cage) or in social novelty preference (novel vs. familiar male mouse) between genotypes.

We evaluated memory performance across different domains. In the fear conditioning test, which assesses associative memory, *Sept3*^−/−^ mice showed slightly higher freezing levels than *Sept3*^+/+^ mice during the conditioning session (Fig. 1f). In the contextual test conducted 24 h after training, freezing was reduced in *Sept3*^−/−^ mice (Fig. 1g). In contrast, no significant differences were observed during the cued test in a novel context (Fig. 1h), or in the remote contextual and cued tests conducted 35 days after training (Fig. 1i, j), indicating a selective impairment in recent contextual memory. In the T-maze forced alternation task, which evaluates spatial working memory, *Sept3*^−/−^ mice exhibited a lower rate of correct responses and shorter latency to reach the goal arm (Fig. 1k, l), while total distance traveled did not differ between genotypes (Fig. 1m).

To assess anxiety-like behavior, we conducted the light/dark transition test, open field test, and elevated plus maze test. In the light/dark transition test, *Sept3*^−/−^ mice showed increased latency to enter the light chamber (Fig. 1n), while time spent in the light area, number of transitions, and distance traveled were comparable between genotypes (Fig. 1o–q). In the open field test, total distance traveled, center time, and vertical activity did not differ significantly between groups (Fig. 1r–t), but stereotypic counts were higher in *Sept3*^−/−^ mice (Fig. 1u). In the elevated plus maze test, no significant differences were observed (Fig. S5). Overall, these results do not reveal strong abnormalities in anxiety-like behavior. In addition, no significant differences were observed between genotypes in the acoustic startle response, prepulse inhibition, forced swim test, or tail suspension test (Fig. S6–S8), indicating no detectable alterations in sensorimotor gating or depression-like behavior in *Sept3*^−/−^ mice.

Taken together, these findings provide the first comprehensive behavioral characterization of male *Sept3*^−/−^ mice, revealing task-specific and context-dependent abnormalities. In the social interaction test, *Sept3*⁻/⁻ mice showed increased interaction in a novel single-chamber test but not in the three-chamber test, indicating a context-dependent dissociation (Fig. 1a–e, S4). Since single-chamber tests allow reciprocal tactile and olfactory exchanges [14], this phenotype may reflect altered responsiveness to direct social cues rather than novelty-induced hyperactivity, as supported by normal initial locomotion in the open field (Fig. 1r). Although such dissociation is rarely reported in genetic models, a similar pattern was observed in chronically isolated mice, which showed hypersociability in a single-chamber test but normal sociability in the three-chamber test [15]. Chronic social isolation involves circuits including the medial prefrontal cortex (mPFC), dorsal raphe nucleus, and ventral hippocampus [16, 17]. While SEPT3 is broadly expressed in the brain [3], its specific role in these regions remains unclear. Our findings may provide a preliminary basis for modeling social isolation-related phenotypes.

In the contextual fear conditioning test, *Sept3*⁻/⁻ mice showed elevated freezing during conditioning (Fig. 1f) but reduced freezing 24 h later (Fig. 1g), indicating a time-dependent impairment in hippocampus-dependent long-term contextual memory. Notably, freezing during the first 1 min after re-exposure was comparable between genotypes, with reduced freezing emerging during 2–4 min (Fig. 1g), consistent with reports that sustained freezing more strongly depends on hippocampal circuits than initial responses [18, 19]. Interestingly, freezing responses at 35 days were comparable between genotypes (Fig. 1i), consistent with the notion that lesions of the hippocampus made after training selectively impair recent memory, whereas remote memory remains intact, as shown in both independent animal cohorts and repeated testing in the same animals [20, 21]. This pattern aligns with our recent finding that *Sept3*⁻/⁻ mice exhibit impaired DG-dependent spatial pattern separation at 1 day but not 2 h after training, rescued by local SEPT3 supplementation [4]. Taken together, these findings suggest that SEPT3 may selectively contribute to recent contextual memory. Cortical engram cells for remote memory can be rapidly generated during initial learning in contextual fear conditioning, even before becoming functionally mature [22]. In this context, the elevated freezing during conditioning (Fig. 1f) indicates that hippocampal encoding was at least partially intact in *Sept3*⁻/⁻ mice, potentially sufficient to initiate systems consolidation. This may explain the preserved remote memory despite impaired recent retrieval. However, because both recent and remote memory were assessed in the same cohort, the possibility that 24-hour re-exposure acted as a reminder cannot be fully excluded, and should be further examined using independent cohorts. Although increased freezing during conditioning may reflect heightened responsiveness to the aversive stimulus, no genotype differences were found in the cued test (Fig. 1h) and hot plate test (Fig. S3), suggesting intact nociception and tone sensitivity.

In the T-maze forced alternation task, *Sept3*⁻/⁻ mice showed reduced correct responses (Fig. 1k, l), indicating a modest deficit in spatial working memory. This task depends on coordinated activity between the hippocampus and mPFC [23]. A similar impairment is seen in heterozygous alpha-Ca^2+^/calmodulin-dependent protein kinase II knockout mice with abnormal mossy fiber (DG–CA3) transmission [24, 25], suggesting that disruption of hippocampal subcircuits can impair task performance. SEPT3, enriched at hippocampal synapses [3, 4], promotes local Ca^2+^ signaling via L-LTP-dependent sER extension into DG spines [4]. Although basal transmission at CA3–CA1 and perforant path–DG synapses is preserved in *Sept3*⁻/⁻ mice [4, 9], further analysis of DG–CA3 and hippocampus–mPFC dynamics may clarify its circuit-level contribution.

In the light/dark transition test, only the latency to enter the light area was increased in *Sept3*⁻/⁻ mice (Fig. 1n–q), possibly reflecting altered approach behavior rather than sustained anxiety [26]. Although this connection remains speculative, theta‑band synchrony between the ventral hippocampus and mPFC has been implicated in approach–avoidance conflict [27], and future studies measuring this synchrony during behavior may clarify whether SEPT3 contributes to this process.

Owing to the widespread expression of SEPT3 and the potential for compensation by other septins [28], the present study cannot yet determine the specific brain regions responsible for the observed behavioral phenotypes. Future studies using region-specific approaches will be necessary to clarify SEPT3’s functional significance.

## Methods

Methods are described in the Supplementary Materials.

## Supplementary Information

Below is the link to the electronic supplementary material.

Supplementary Material 1

## Data Availability

No datasets were generated or analysed during the current study.

## Declarations

### Abbreviations

SEPT3 septin 3.

L-LTP late-phase long-term potentiation.

sER smooth endoplasmic reticulum.

DG dentate gyrus.

GTP guanosine triphosphate.

CA1 cornu ammonis 1.

mPFC medial prefrontal cortex.
