# Supplementary material for: Comprehensive behavioral phenotyping of male Septin 3-deficient mice reveals task-specific abnormalities
Source: Mol Brain. 2025 Aug 22;18:71. doi: 10.1186/s13041-025-01243-5 (PMC12374300; doi:10.1186/s13041-025-01243-5)
Supplement: Supplementary file 1 — Supplementary Material 1 [file 13041_2025_1243_MOESM1_ESM.pdf]

Supplementary Materials

Methods

Animals and experimental design

Male *Sept3* knockout (*Sept3*$^{-/-}$) and wild-type (*Sept3*$^{+/+}$) littermates were generated by intercrossing *Sept3*$^{+/-}$ heterozygotes. Mice were housed in same-sex groups under standard laboratory conditions with a 12-h light/dark cycle (lights on at 7:00) and had ad libitum access to food and water. To minimize potential environmental variation, mice used in behavioral experiments were bred and reared under identical conditions. The age at testing is provided in the figure legends.

All behavioral testing was conducted during the light phase. Behavioral assessments were performed in the following order: general health and neuromuscular function screening (including body weight and temperature measurements, wire hang test, and grip strength test); light/dark transition test; open field test; elevated plus maze test; rotarod test; hot plate test; single-chamber social interaction test; three-chamber social interaction test; acoustic startle response and prepulse inhibition test; Porsolt forced swim test; T-maze forced alternation test; tail suspension test; and contextual and cued fear conditioning test.

All procedures were approved by the Institutional Animal Care and Use Committees of the National Institute for Physiological Sciences and Nagoya University, and were conducted in compliance with relevant institutional guidelines.

Wire hang test and grip strength test

For the wire hang test, a wire grid was used. Mice were placed on top and rotated gently to an inverted position. Latency to fall was recorded. Forelimb grip strength was measured by pulling a mouse by the tail while its forepaws held onto a wire grid attached to a spring balance. The tensile force (N) when the mouse released the grid was measured three times, and the greatest value was analysed.

Light/dark transition test

The apparatus (21 × 41 × 25 cm) consisted of connected light and dark chambers with contrasting illumination (390 lux vs. 2 lux). Each mouse was initially placed in the dark compartment and allowed to explore freely for 10 minutes. Latency until the first entry into the light chamber, time spent in each chamber, number of transitions, and total distance traveled in each chamber were recorded using automated tracking.

Open field test

A square open field chamber (40 × 40 × 30 cm) illuminated at approximately 100 lux was used. Each

mouse was placed in the center and allowed to explore freely for 120 minutes. Total distance traveled, time spent in the central area (20 × 30 cm), rearing frequency, and stereotypic movements were recorded automatically using a tracking system.

Elevated plus maze test

A plus-shaped maze elevated 55 cm above the floor was used, consisting of two open arms (25 × 5 cm) and two closed arms (25 × 5 cm with 15 cm-high walls) and illuminated at 100 lux. Mice were placed at the center and allowed to explore freely for 10 minutes. Total distance traveled, as well as the number of entries into and time spent in the open and closed arms, were automatically recorded.

Rotarod test

The rod diameter was 3 cm, and the rotation speed increased gradually from 4 to 40 rpm over 5 minutes. Mice were placed on the rod for 6 trials across two consecutive days. Latency to fall was automatically recorded for each trial.

Hot plate test

A 55 °C hot plate was used, and latency to the initial nociceptive reaction (e.g., paw licking or jumping) was recorded with a set cut-off time to avoid injury.

Single-chamber social interaction test

Each mouse was placed with an unfamiliar mouse in the novel chamber (40 × 40 × 30 cm) and allowed to interact freely for 10 minutes. The number of contacts, duration of social interaction, and total distance traveled were recorded using automated tracking.

Three-chamber social interaction test

A three-chamber apparatus (20 × 40 × 22 cm) separated by transparent partitions with 5 × 3 cm openings was used. Each side chamber contained a cylindrical wire cage (9 cm diameter) that allowed nose contact with the enclosed unfamiliar stimulus mouse. The subject mouse was placed in the center chamber and allowed to explore freely for two 10-minute sessions: the sociability test, with one unfamiliar mouse placed in one side cage; and the social novelty preference test, with an additional unfamiliar mouse introduced in the opposite cage. Time spent in each chamber and time within proximity (within 5 cm) of each cage were recorded using automated tracking.

Acoustic startle response and prepulse inhibition test

A startle response measurement chamber with a restraining cylinder and 70 dB background white noise was used. After 10 minutes, startle responses to 40 ms white noise pulses at 110 or 120 dB were recorded. Each session consisted of six blocks of six trials presented in pseudorandom order, including trials with a prepulse stimulus (74 or 78 dB white noise) delivered 100 ms before the startle stimulus and trials without a prepulse. The average inter-trial interval was 15 seconds.

Porsolt forced swim test

An acrylic cylinder (10 cm diameter) filled with water to a depth of 7.5 cm at 23 °C was used. Each mouse was placed in the water for up to 10 minutes. Duration of immobility was recorded automatically using a tracking system.

T-maze forced alternation test

A fully automated T-maze apparatus was constructed with white plastic walls 25 cm high and was divided into six areas by automated doors. The maze included the start area, two connecting passages, the T-junction end (13 × 24 cm), and two arms (A1 and A2; 11.5 × 20.5 cm each). Pellet dispensers were installed at the ends of the arms to deliver food rewards, and sensors detected whether the pellet was consumed. Mice were food-restricted for more than one week prior to testing to maintain body weight below 85%, and this restriction was continued during testing. Following a 30-minute habituation and reward retrieval training session with all doors open, mice were tested on the forced alternation task, which was conducted in 10 trials per day over 8 consecutive days. In each trial, mice were first forced into either A1 or A2 to receive a food pellet. After the mouse consumed the pellet or 30 seconds elapsed, the doors opened, allowing the mouse to return to the start area via the passage. Three seconds after returning to the start area, the doors reopened, permitting free choice of either arm. The rewarded arm was the one opposite to the arm chosen in the forced run; choosing the previously visited arm resulted in no reward and confinement for 10 seconds. The initial forced arm was determined in a pseudorandom sequence. Correct choice rate, total session duration, and total distance traveled were recorded automatically.

Tail suspension test

Each mouse was suspended by the tail at a height of 30 cm for 10 minutes. The movement was recorded for analysis.

Contextual and cued fear conditioning test

Two distinct chambers were used: a rectangular acrylic box (26 × 34 × 29 cm) with a metal grid floor

(0.2 cm diameter rods at 0.5 cm intervals), transparent front and back walls, white side walls, and illuminated at 100 lux; and a white acrylic triangular prism chamber (33 × 29 × 32 cm) illuminated at 30 lux. On the first day, mice were placed in the rectangular chamber for 8 minutes. At 2, 4, and 6 minutes after placement, a 30-second 55 dB white noise cue was presented, with a 0.3 mA footshock delivered during the final 2 seconds of each cue. On the following day and 35 days later, the same mice underwent context and cued tests. For the context test, mice were returned to the same rectangular chamber for 5 minutes without any cues or shocks. For the cued test, mice were placed in the triangular chamber for 6 minutes; the same white noise cue was presented from 3 to 6 minutes. Immobility duration and total distance traveled were recorded automatically during both tests.

Supplementary Figures

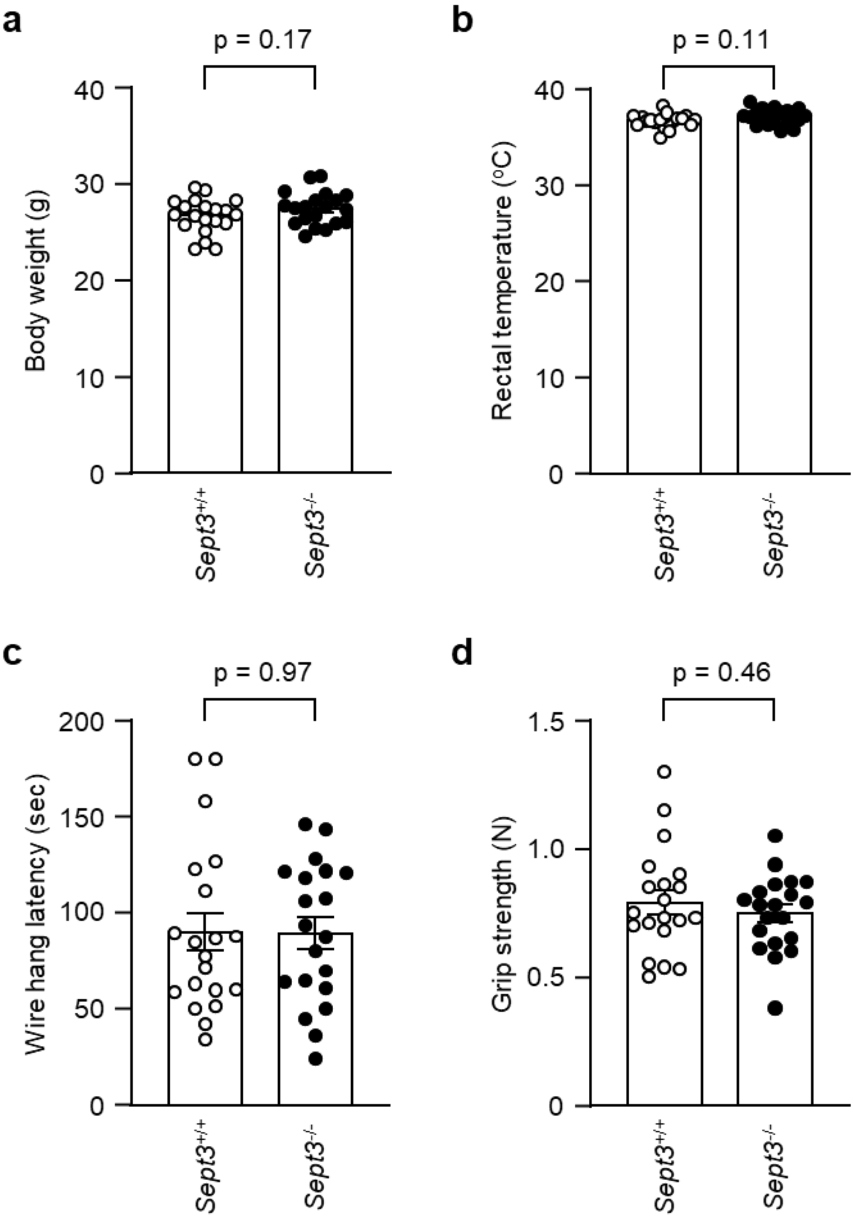

Figure S1

General health and neuromuscular function.

a, Body weight. **b**, Rectal temperature. **c**, Latency to fall in the wire hang test. **d**, Forelimb grip strength. n = 20 (*Sept3*$^{+/+}$) and n = 20 (*Sept3*$^{-/-}$) 10–12-week-old male mice; two-tailed unpaired *t* test. Data are mean ± SEM.

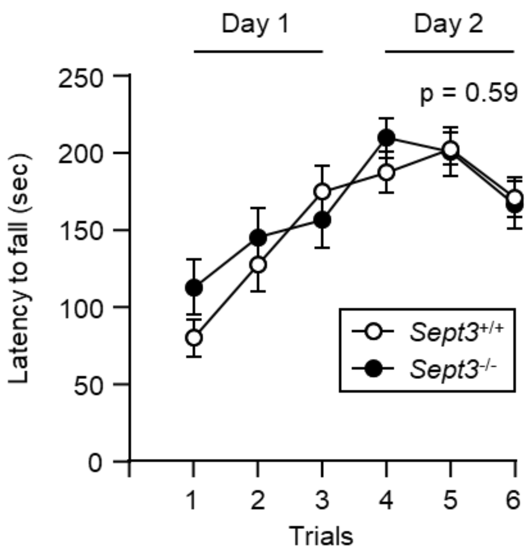

Figure S2

Rotarod test.

Latency to fall from an accelerating rotarod across repeated trials [$F_{1,38} = 0.29$, $p = 0.59$, genotype × time interaction, $F_{5,190} = 1.20$, $p = 0.31$]. n = 20 (*Sept3*$^{+/+}$) and n = 20 (*Sept3*$^{-/-}$) 12–14-week-old male mice; two-way repeated measures ANOVA. Data are mean ± SEM.

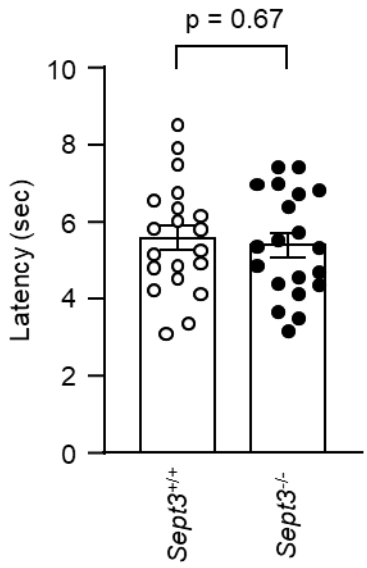

Figure S3
Hot plate test.

Latency to respond to a noxious thermal stimulus. n = 20 (*Sept3*$^{+/+}$) and n = 20 (*Sept3*$^{-/-}$) 12–14-week-old male mice; two-tailed unpaired *t* test. Data are mean ± SEM.

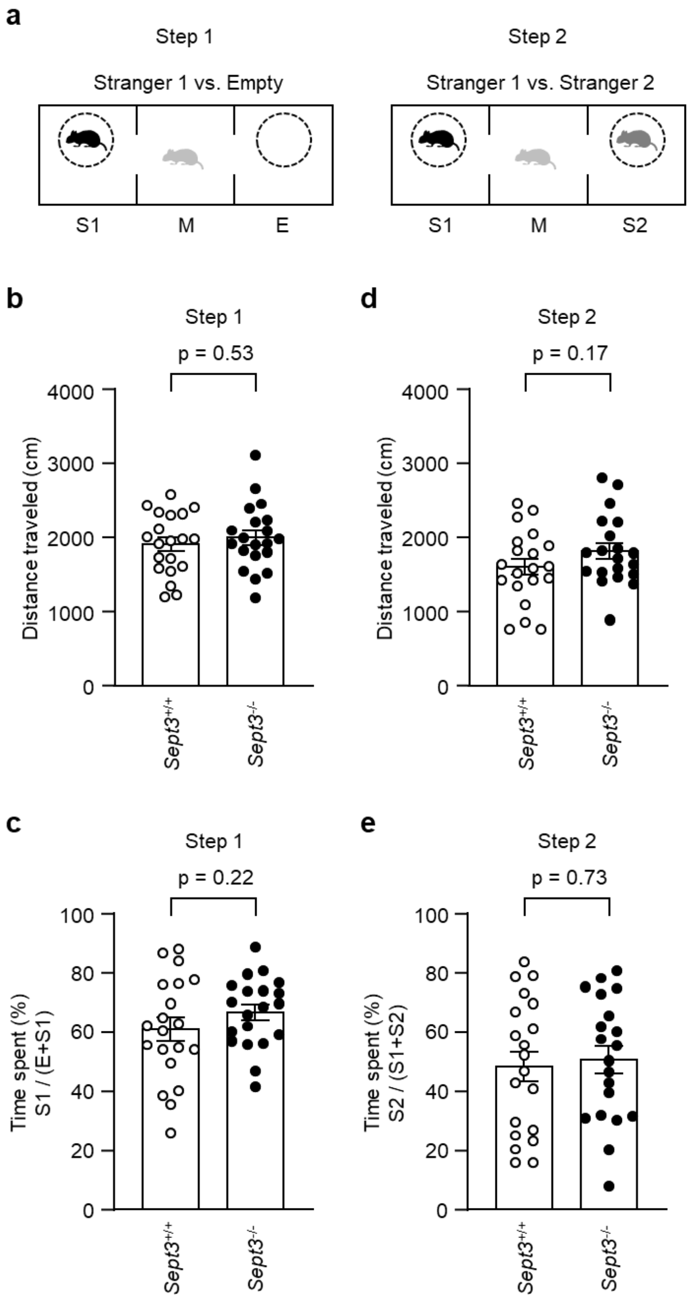

Figure S4

Three-chamber social interaction test.

a, Schematic of the two-step testing protocol: Step 1, sociability test (Stranger 1 vs empty cage); Step 2, social novelty preference test (Stranger 1 vs Stranger 2). **b**, **d**, Total distance traveled during Step 1 (**b**) and Step 2 (**d**). **c**, Time spent in proximity to Stranger 1 during Step 1, expressed as a percentage of total time near both cages. **e**, Time spent in proximity to Stranger 2 during Step 2, expressed as a percentage of total time near both stranger cages. n = 20 (*Sept3*$^{+/+}$) and n = 20 (*Sept3*$^{-/-}$) 13–15-week-old male mice; two-tailed unpaired *t* test. Data are mean ± SEM.

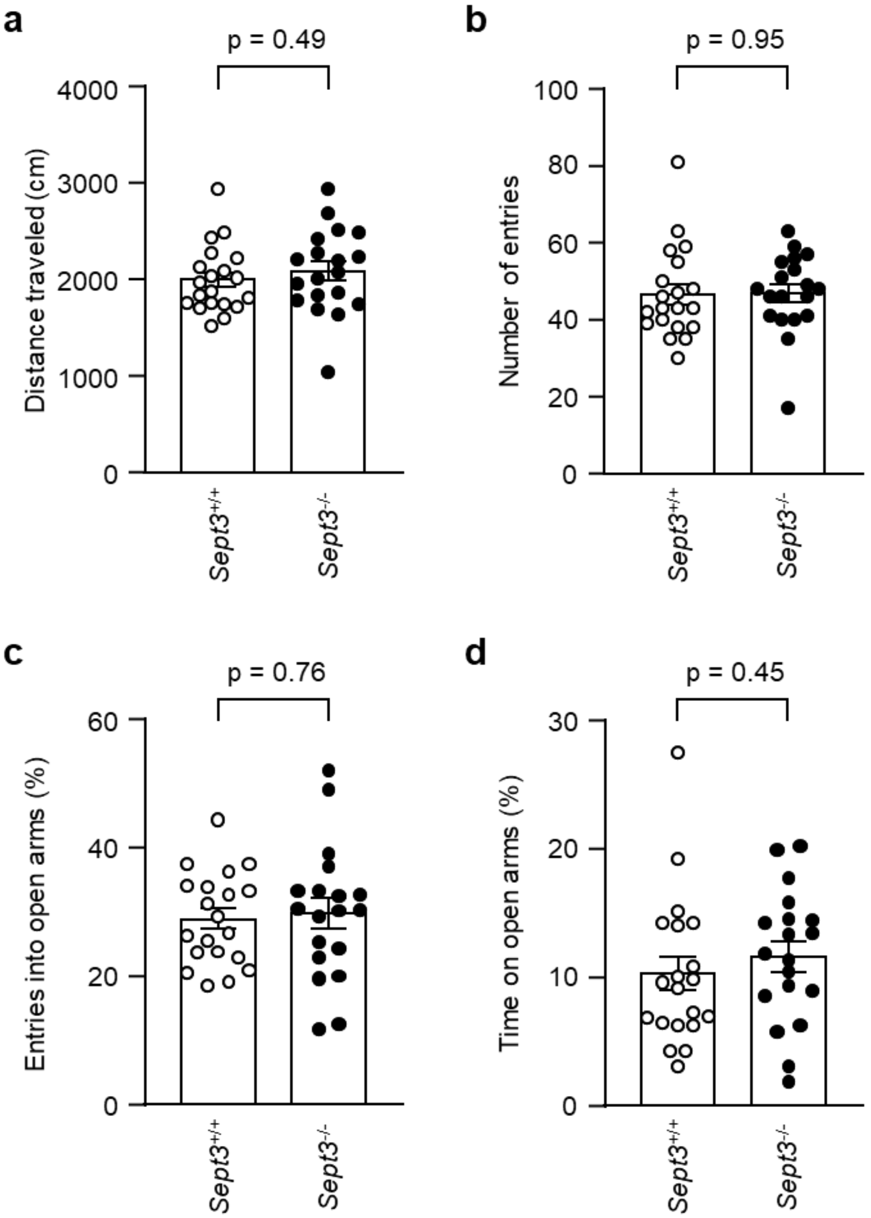

Figure S5

Elevated plus maze test.

a, Total distance traveled. **b**, Total number of arm entries. **c**, Percentage of entries into open arms. **d**, Percentage of time spent in open arms. n = 20 (*Sept3*$^{+/+}$) and n = 19 (*Sept3*$^{-/-}$) 11–13-week-old male mice; two-tailed unpaired *t* test. Data are mean ± SEM.

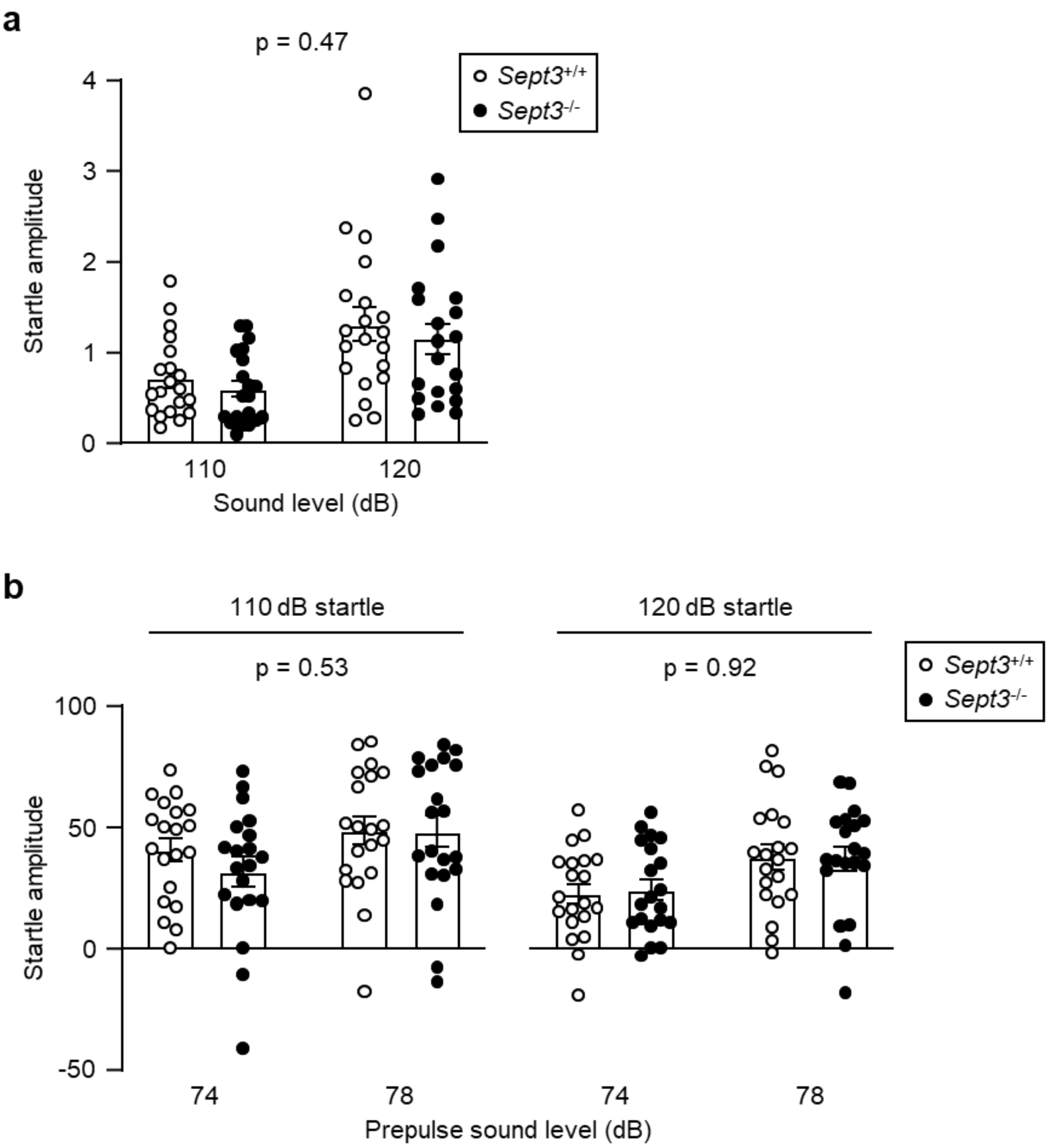

Figure S6

Acoustic startle response and prepulse inhibition test.

a, Startle amplitude (arbitrary unit) to 110 dB and 120 dB acoustic stimuli [$F_{1,38} = 0.52$, $p = 0.47$, genotype × time interaction, $F_{1,38} = 0.076$, $p = 0.78$]. **b**, Percent reduction of startle amplitude in the presence of a preceding acoustic stimulus (prepulse) [110 dB, $F_{1,38} = 0.40$, $p = 0.53$, genotype × time interaction, $F_{1,38} = 1.56$, $p = 0.22$, 120 dB, $F_{1,38} = 0.010$, $p = 0.92$, genotype × time interaction, $F_{1,38} = 0.11$, $p = 0.74$]. n = 20 (*Sept3*$^{+/+}$) and n = 20 (*Sept3*$^{-/-}$) 14–16-week-old male mice; two-way repeated measures ANOVA. Data are mean ± SEM.

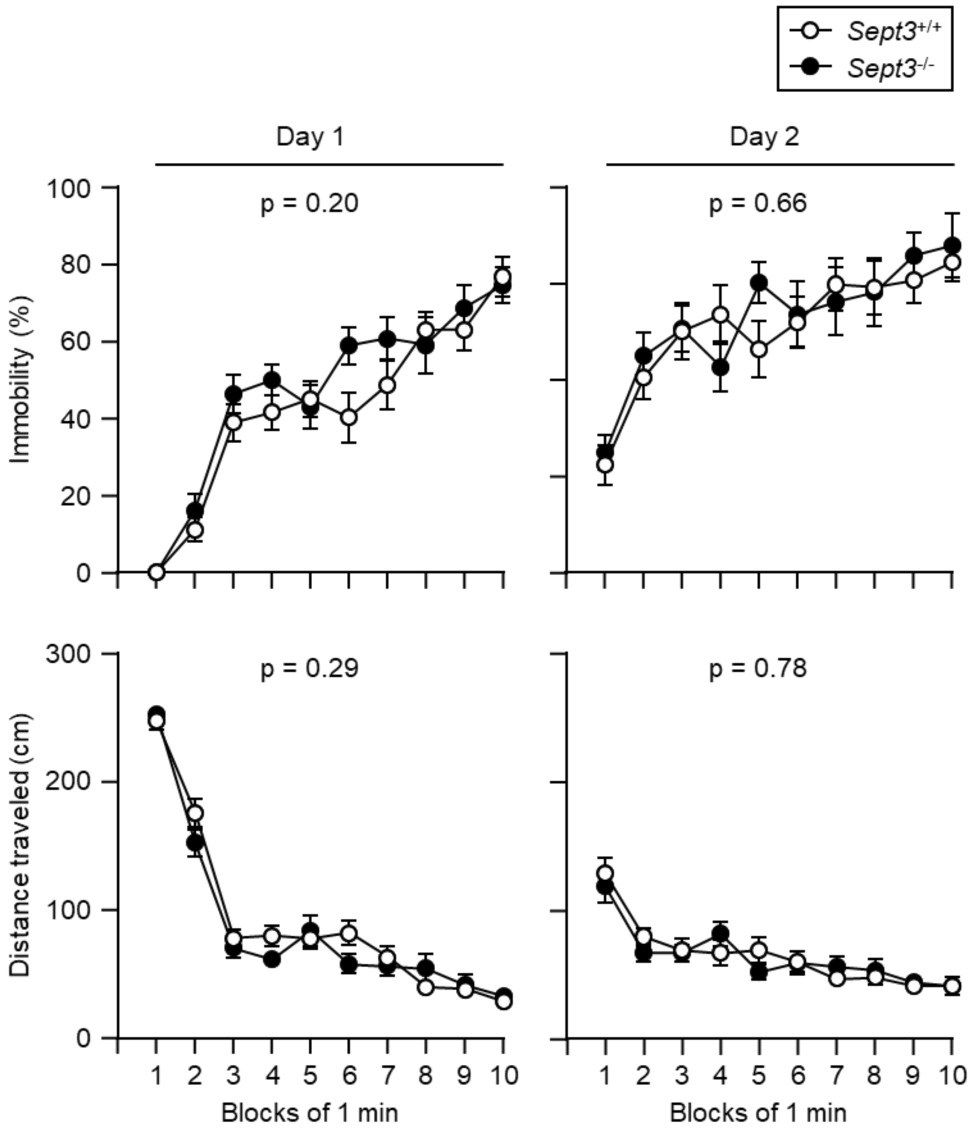

Figure S7

Porsolt forced swim test.

Top, Percent immobility on Day 1 and Day 2 [Day 1, $F_{1,38} = 1.72$, $p = 0.20$, genotype × time interaction, $F_{9,342} = 1.28$, $p = 0.25$, Day 2, $F_{1,38} = 0.19$, $p = 0.66$, genotype × time interaction, $F_{9,342} = 0.79$, $p = 0.62$]. Bottom, Distance traveled in water on Day 1 and Day 2 [Day 1, $F_{1,38} = 1.17$, $p = 0.29$, genotype × time interaction, $F_{9,342} = 1.59$, $p = 0.12$, Day 2, $F_{1,38} = 0.082$, $p = 0.78$, genotype × time interaction, $F_{9,342} = 0.79$, $p = 0.63$]. n = 20 (*Sept3*$^{+/+}$) and n = 20 (*Sept3*$^{-/-}$) 15–17-week-old male mice; two-way repeated measures ANOVA. Data are mean ± SEM.

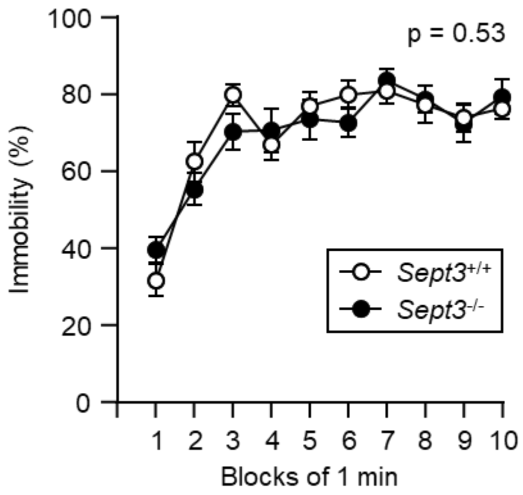

Figure S8

Tail suspension test.

Percent immobility across 1-minute blocks during tail suspension [$F_{1,37} = 0.41$, $p = 0.53$, genotype × time interaction, $F_{9,333} = 0.94$, $p = 0.49$]. n = 20 (*Sept3*$^{+/+}$) and n = 19 (*Sept3*$^{-/-}$) 37–39-week-old male mice; two-way repeated measures ANOVA. Data are mean ± SEM.
